# Noticeable Improvement

**Published:** 1917-02

**Authors:** 


### Noticeable Improvement.

The editor trusts that the readers of the TEXAS MEDICAL JOURNAL will not think that to call attention occasionally to its merits as a medical publication can be called boasting, although it may possibly be termed boosting. Since so few readers make critical comparisons without being invited to do so, they will all probably excuse this invitation to compare the contents of this JOURNAL with any other sold at the same subscription price. There are larger journals published, more attractive looking journals perhaps, but they are usually sold at from two to five times the price of the TEXAS MEDICAL JOURNAL, and most of them contain a disproportionate amount of advertising.

Many readers write that they like the TEXAS MEDICAL JOURNAL because it is the journal of all journals that seems to be edited for the busy practitioner rather than for the purpose of allowing some medical wise-acres to display their technical learning. It is true that of necessity many articles dealing with medical and surgical questions are from necessity couched in professional terms, but it is nearly always impossible for a writer on such themes to make himself intelligible in the language of the laity. Therefore he must use the technical language of the profession. But most writers and contributors to this JOURNAL try to say what they have to say in the plainest terms and in the fewest words possible.

Then, in variety of subjects discussed, it is doubtful if any other journal excels this one. The editor studies closely the things that are going on in medical science, and when a thing of interest is noted the best authorities on that subject are invited to write upon it. Up-to-dateness is absolutely necessary in a medical publication.

Again, those subjects are given most space that are of most interest to this section of the country, where the TEXAS MEDICAL JOURNAL circulates most largely. The South and Southwest have their own problems that differ largely from those of the North and the East. The physicians that would keep informed with the things that concern them directly cannot do so through a Northern journal any better than a Southern farmer can learn how to grow cotton from a Northern farm paper.

The editor believes that the TEXAS MEDICAL JOURNAL grows better every month,—at least an effort is being made to improve it,—but if you think it is not and can suggest in what way it can be made better, please consider this a personal appeal to you to sit down and write the editor whatever suggestions may occur to you. Above all things else, it is desired that this be regarded as your special journal in which you are enough interested to feel free to make whatever suggestions you may wish. Will you do it?

